# Endoscopic removal of ectopic sinonasal teeth: a systematic review

**DOI:** 10.1186/s40463-019-0353-8

**Published:** 2019-07-05

**Authors:** Marc Levin, Doron D. Sommer

**Affiliations:** 1 Michael G. DeGroote School of Medicine, Michael G. DeGroote Centre for Learning and Discovery, McMaster University, 1280 Main Street West, 3104, Hamilton, ON L8S 4K1 Canada; 2 Department of Surgery, Division of Otolaryngology - Head and Neck Surgery, Hamilton Health Sciences, McMaster University, Hamilton, Canada

**Keywords:** Endoscopic sinus surgery, Ectopic sinonasal teeth, Endoscopic teeth removal, Caldwell-Luc, Chronic rhinosinusitis

## Abstract

**Introduction:**

Ectopic sinonasal teeth are uncommon. The classic approach to removal of such foreign bodies was the Caldwell-Luc. In recent years however, endoscopic approaches have become increasingly utilized. Despite this, there is a dearth of literature and consensus regarding the endoscopic removal of ectopic sinonasal teeth. As such, we conducted a systematic review on all cases of endoscopic removal of ectopic sinonasal teeth in the literature. With an understanding of the literature, clinical and technical decision making for patients with this pathology may be elucidated.

**Methods:**

Systematic review of the Ovid Medline, EMBASE Classic and Pubmed databases were conducted using PRISMA guidelines.

**Results:**

Our search identified 100 articles. Final inclusion consisted of 23 studies with a total of 27 patient cases. The majority of the patients included were male (70.4%) with a mean age of 27.06 years. Patients presented with a multitude of symptoms, with nasal obstruction (48.14%), rhinorrhea (22.2%), facial pain (22.2%) and epistaxis (22.2%) being most common. Surgeons mostly reported using a 0° endoscope (22.2%) and performing a maxillary antrostomy/uncinectomy (37%) and simple extraction under general anesthetic (41%).

**Conclusions:**

This systematic review analyzed important epidemiological, clinical and technical information regarding patients with endoscopic removal of sinonasal ectopic teeth. Further research is needed to promote implementation of such data into clinical practice.

## Introduction

Sinonasal ectopic teeth are uncommon. The etiology of teeth in the maxillary sinus is most commonly secondary to trauma, including iatrogenic/dental procedures [1, 2]. .Supernumerary teeth can also erupt idiopathically into the nasal cavity [3]. In the past, Caldwell-Luc (trans-antral) type approaches were most commonly performed to remove foreign bodies from the maxillary sinus [4–10].

In recent years, endoscopic removal of maxillary sinus foreign bodies has become an often preferred technique [11–13]. Compared to the Caldwell-Luc procedure, the endoscopic removal of maxillary sinus foreign bodies can reduce perioperative morbidity and complications and shorten operating times [14, 15]. Despite widespread applicability of endoscopic techniques, surgeons are still reporting using the trans-antral approach for maxillary sinus ectopic tooth removal [16–18]. This can be combined with endoscopic techniques both as a second portal and to achieve more lateral/anterior access, although this may also be achieved by endoscopic access anterior to the lacrimal duct. Chronic maxillary rhinosinusitis caused by various foreign bodies such as dental implants [19], dental impressions [20] and even toothpicks [21] among others have been described. Despite the benefits of the endoscopic approach, very few case reports describe the endoscopic removal of ectopic maxillary sinus or nasal cavity teeth causing chronic rhinosinusitis or nasal obstruction. While there have previously been reviews regarding literature on ectopic maxillary sinus teeth [22], none of these have specifically focused on endoscopic removal of paranasal sinus and nasal cavity teeth.

It is thus beneficial to highlight the role of the endoscopic approach for removal of ectopic paranasal sinus teeth through this systematic review. Hence, this review aims to summarize the literature on the endoscopic removal of sinonasal ectopic teeth. By systematically reviewing the literature and understanding these technical and clinical details, otolaryngologists and oral maxillofacial surgeons may be able to better diagnosis and stratify patients and select their management method from an evidence-based perspective.

## Methods

### Study eligibility

Articles in the medical, surgical and dental/oral maxillofacial surgery specific literature describing endoscopic removal of ectopic paranasal sinus/nasal cavity teeth were included. For this systematic review, we defined ‘endoscopic removal’ as the surgical removal of a paranasal sinus tooth using a trans-nasal endoscopic approach. We defined ‘paranasal sinus/nasal cavity’ as either the maxillary, frontal, sphenoid or ethmoid sinuses and the nasal cavity space. Finally, we defined ‘ectopic tooth’ as any ectopic, supernumerary or impacted teeth found in the paranasal sinuses/nasal cavity. Studies assessing patients with other paranasal foreign bodies, such as dental implants, were excluded from this review. Studies assessing patients with ectopic teeth in anatomical areas other than the paranasal sinuses/nasal cavity were excluded. Original research studies published in English in peer reviewed journals were included. Randomized controlled trials, observational, cohort, case-control, case series, case reports, cross sectional were incorporated in this review. Unpublished abstracts, conference posters, reviews, letters to editors, editorials were excluded from our search. This review was completed in accordance with PRISMA guidelines [23].

### Databased searched

One author conducted a search in Ovid Medline, EMBASE Classic and Pubmed databases. Additional searches in Google Scholar and in the references of selected articles were completed to ensure all possible relevant articles were included. This search was completed on January 16th, 2019 and included all articles since the inception dates of the databases.

### Search

Medical subject headings (MeSH) terms used in the search included: ‘nasal cavity’, ‘sinonasal’, ‘maxillary sinus’, ‘paranasal sinus’, ‘tooth eruption’, ‘tooth ectopic’, ‘tooth supernumerary’, ‘dentigerous cyst’, ‘tooth impacted’, ‘endoscopic sinus surgery’, ‘functional endoscopic sinus surgery’, ‘sinus surgery’, maxillary antrum’, ‘tooth’. Titles of articles resulting from these searchers and abstracts were then reviewed. Any article that fit the eligibility criteria were selected for full-text review. Two authors (ML and DDS) assessed each article yielded from the search for inclusion, according to the previously described eligibility criteria. Any disagreements in article selection, were discussed by both authors until an agreement was reached.

### Data extraction

After both authors (ML and DDS) agreed upon all articles that were to be included in the review, data were extracted from the articles. Data including demographic data such as patient age and patient gender were extracted. Clinical data such as tooth type, tooth location and clinical presentation were also extracted. Finally, information regarding surgical removal technique and clinical outcome of the surgery was extracted from the included studies.

### Data analysis

Descriptive results were quantified and analyzed using Microsoft Excel.

## Results

**Fig. 1:**
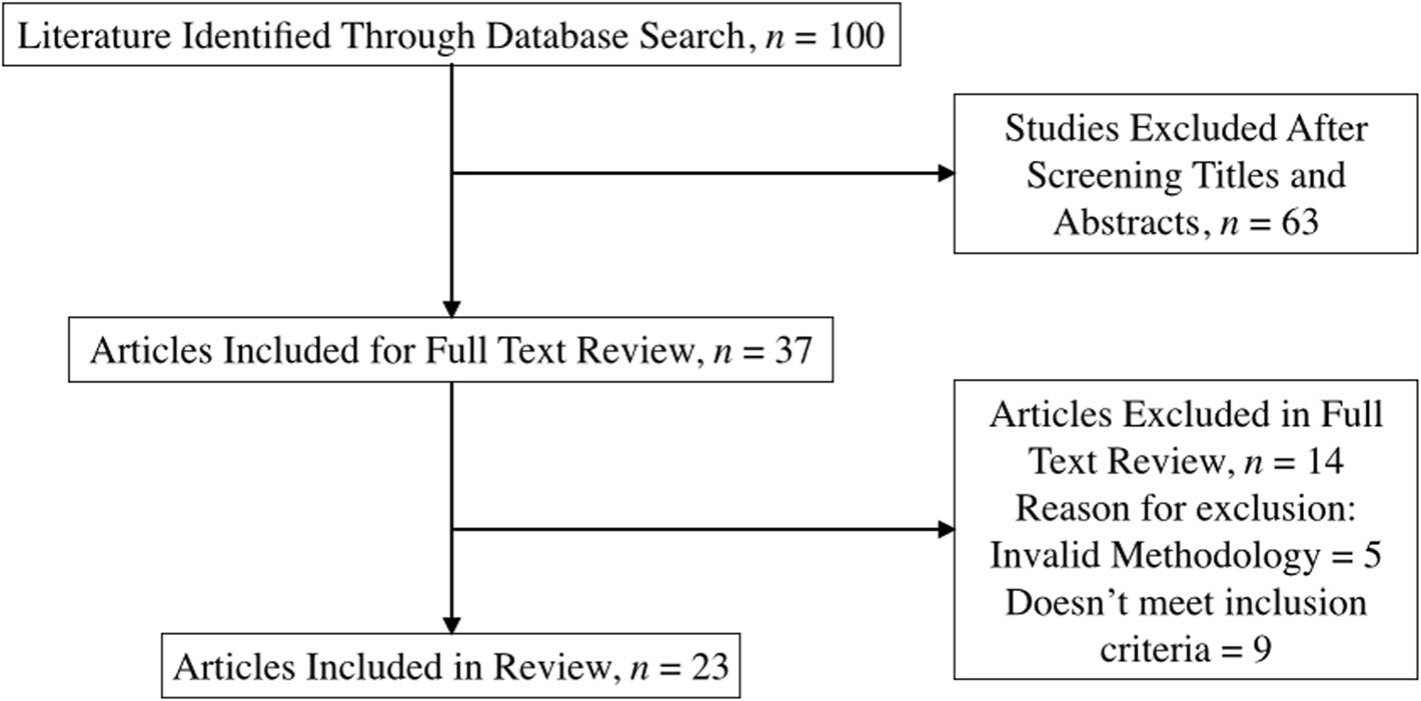
PRISMA Flowchart

Our search identified 100 articles (Fig. 1). Final inclusion consisted of 23 studies (Table 1). These papers included studies identifying the endoscopic removal of teeth in the nasal cavity as well as the maxillary sinus. The total number of patient cases extracted from included studies was 27. The majority of patients included in this review were male (70.4%) in comparison to female (29.6%). The mean age of patients included in the review was 27.06 years. All patients underwent endoscopic removal of their ectopic tooth. 13 teeth (48.14%) were present in the maxillary sinus and 14 (51.86%) were in the nasal cavity.

**Table 1:** Articles Included In Systematic Review

Author	Year	Patient Age (Years)	Patient Gender	Tooth Type	Tooth Location	Clinical Presentation	Pre-Operative Endoscopic And Imaging Findings	Imaging Modality Used	Removal Technique	Post-Operative Endoscopic And Imaging Findings	Outcome	Time Of Post-Operative Follow-Up
Al Nashawany Et Al. [24]	2014	50	Female	Tooth Root	Maxillary Antrum	Nasal Regurgitation And Recurrent Maxillary Sinusitis	Endoscopic: Pus In The Left Middle Meatus, Edematous Polypoidal Mucosa. CT Scan: Opacification Of Maxillary Antrum	CT Scan Paranasal Sinuses	Endoscopic	CT Scan: Clear Maxillary Antrum	Complete Resolution Of Symptoms	N/A
Hasbini Et Al. [25]	2001	21	Male	Molar Tooth	Maxillary Sinus	Nasal Obstruction, Headache And Hyoosmia	Endoscopic: Medial Bulge Of The Lateral Nasal Wall. CT Scan: Opacification Of Entire Maxillary Sinus	CT Scan Paranasal Sinuses	Endoscopic	N/A	Complete Resolution Of Symptoms	6 Months
Di Pasquale And Shermetaro [26]	2006	14	Female	Molar Tooth	Maxillary Sinus	None	Endoscopic: Unremarkable. CT Scan: Entire Maxillary Sinus Opacification	Panoramic X-Rays And CT Scan Paranasal Sinuses	Endoscopic	N/A	N/A	2 Years
Saleem Et Al. [27]	2010	45	Male	Ectopic Tooth	Maxillary Sinus	Hemoptysis	Endoscopic: Bilateral Osteomeatal Erythema. CT Scan: Complete Opacification Of Maxillary Antrum And Widening Of Ostium. Mucosal Thickening Of Left Ethmoidal Cells.	CT Scan Paranasal Sinuses	Endoscopic	N/A	Complete Resolution Of Symptoms	10 Days
Chandrasena Et Al. [12]	2010	N/A	N/A	Tooth Root	Maxillary Sinus	N/A	N/A	Orthopantogram And CT Scan Paranasal Sinuses	Endoscopic	N/A	N/A	N/A
Viterbo Et Al. [28]	2013	29	Male	Ectopic Tooth	Maxillary Sinus	N/A	N/A	Panoramic X-Rays And CT Scan Maxillofacial Region	Endoscopic	N/A	N/A	1 Year
Oinam Et Al. [14]	2016	40	Male	Impacted Maxillary Third Molar Tooth	Maxillary Sinus	None	Radio-Opaque/Dense Shadow In Right Maxillary Sinus	X-Ray Paranasal Sinuses And CT Scan Paranasal Sinuses	Endoscopic	X-Ray: No Abnormalities	N/A	2 Weeks
Iwai Et Al. [29]	2012	38	Male	Molar Tooth	Maxillary Sinus	N/A	N/A	N/A	Endoscopic	N/A	N/A	N/A
Clementini Et Al. [30]	2012	9	N/A	Ectopic Tooth	Maxillary Sinus + Nasal Floor	None	N/A	Orthopantogram And CT Scan Paranasal Sinuses	Endoscopic	N/A	N/A	1 Month
Nisa And Giger [31]	2011	30	Male	Ectopic Tooth	Maxillary Sinus + Osteomeatal Complex	Maxillary And Dental Pain And Purulent Oral Discharge	Endoscopic: Purulence From Opening In Gum. CT Scan: Large Mucocele In Osteomeatal Complex And Destruction Of Bony Floor Of Maxillary Sinus	CT Scan Paranasal Sinuses	Endoscopic	N/A	Complete Resolution Of Symptoms	8 Months
Chen Et Al. [32]	2002	8	Male	Supernumerary Tooth	Nasal Cavity	Nasal Obstruction	Endoscopic: White Mass With Granulation Tissue In Nasal Cavity. CT Scan: Mass With Soft Tissue Surrounding	CT Scan Paranasal Sinuses	Endoscopic	N/A	Complete Resolution Of Symptoms	2 Weeks
Chen Et Al. [32]	2002	7	Female	Supernumerary Tooth	Nasal Cavity	Nasal Obstruction And Purulent Discharge	Endoscopic: White Mass With Granulation Tissue Surrounding. CT Scan: Mass With Soft Tissue Surrounding.	CT Scan Paranasal Sinuses	Endoscopic	N/A	Complete Resolution Of Symptoms	N/A
Chen Et Al. [32]	2002	59	Female	Supernumerary Tooth	Nasal Cavity	Nasal Obstruction	Endoscopic: White Mass Surrounded By Black-Yellow Substance. CT Scan: Irregular Punctate Radiopacities Posterior To Toothlike Structure.	CT Scan Paranasal Sinuses	Endoscopic	N/A	Complete Resolution Of Symptoms	3 Weeks
Aoki Et Al. [33]	2003	7	Female	Tooth	Nasal Cavity	Nasal Obstruction And Epistaxis	N/A	Panoramic X-Rays And CT Scan Paranasal Sinuses	Endoscopic	N/A	Complete Resolution Of Symptoms	N/A
Kim Et Al. [34]	2003	12	Male	Supernumerary Tooth	Nasal Cavity	None	Endoscopic: No Cystic Or Inflammatory Changes Noted	Panoramic X-Rays	Endoscopic	N/A	N/A	2 Years
Lin Et Al. [35]	2004	16	Female	Aberrant Tooth	Nasal Cavity	Nasal Obstruction Epistaxis	Endoscopic: White, Hard Mass Erupting From Nasal Cavity Floor	CT Scan Paranasal Sinuses	Endoscopic	N/A	Complete Resolution Of Symptoms	2 Years
Lin Et Al. [35]	2004	21	Male	Tooth	Nasal Cavity	Nasal Obstruction And Purulent Rhinorrhea	Endoscopic: Granulomatous Lesion With Nectrotic Tissue In Nasal Cavity. CT Scan: No Destruction Of Structures And Air-Fluid Level In Maxillary Sinus.	Water’s View X-Ray. CT Scan Paranasal Sinuses	Endoscopic	N/A	Complete Resolution Of Symptoms	N/A
Lin Et Al. [35]	2004	6	Female	Tooth	Nasal Cavity	Right Nasal Obstruction	Endoscopic: Hard Mass From Base Of Right Nasal Cavity.	Water’s View X-Ray	Endoscopic	N/A	Complete Resolution Of Symptoms	N/A
Lee [36]	2006	61	Male	Supernumerary Tooth	Nasal Cavity	Foul Odor In Nose And Nasal Obstruction	Endoscopic: White Mass With Yellow-Brown Color Crust In Nasal Floor. CT Scan: Cone-Shaped High-Density Mass	CT Scan Paranasal Sinuses	Endoscopic	N/A	Complete Resolution Of Symptoms	N/A
Verma Et Al. [37]	2012	3.5	Male	Ectopic Tooth	Nasal Cavity	Recurrent Epistaxis	Endoscopic: Purulent Discharge In Floor Of Right Nasal Cavity With Thickened Nasal Vestibule. CT Scan: Dense Shadow In Floor Of Nasal Cavity.	CT Scan Paranasal Sinuses	Endoscopic	N/A	Complete Resolution Of Symptoms	N/A
Janardhan Et Al. [38]	2013	30	Male	Supernumerary Ectopic Tooth	Nasal Cavity	Nasl Obstruction, Recurrent Epistaxis, And Nasal Discharge	Endoscopic: Yellow –Black Mass In Nasal Cavity.	X-Ray Paranasal Sinuses	Endoscopic	N/A	N/A	N/A
Koosha Et Al. [39]	2014	33	Female	Tooth	Nasal Cavity	Epistaxis And Foul Smelling Nasal Discharge	Endoscopic: Calcified, Brown Rhinolith	Occipitomental View X-Ray	Endoscopic	X-Ray: No Abnormalities	Complete Resolution Of Symptoms	6 Weeks
Sanei-Moghaddam Et Al. [40]	2009	30	Male	Supernumerary Tooth	Nasal Cavity	Unilateral Nasal Obstruction And Blood Stained Discharge	CT Scan: Tooth Surrounded By Granulation Tissue And Rhinolith	Orthopantogram And CT Paranasal Sinuses	Endoscopic	N/A	Complete Resolution Of Symptoms	N/A
Larin Et Al. [41]	2013	N/A	N/A	Impacted Wisdom Tooth	Maxillary Sinus	Maxillary Sinus Pain And Nasal Congestion	N/A	CT Scan Paranasal Sinuses	Endoscopic	N/A	Complete Resolution Of Symptoms	N/A
Aydin Et Al. [42]	2016	21	Male	Ectopic Tooth	Maxillary Sinus	Facial Pain And Pressure	Endoscopic: Normal Nasal Mucosa. Ct Scan: Completely Opacified Maxillary Sinnus	Orthopantrogram And CT Scan Paranasal Sinuses	Endoscopic	N/A	Complete Resolution Of Symptoms	N/A
Ohki [43]	2012	37	Male	Impacted Tooth	Maxillary Sinus	Nasal Discharge And Odontalgia	CT Scan: Cystic And Calcificated Lesion	Water’s View X-Ray And CT Scan Paranasal Sinuses	Endoscopic	N/A	Complete Resolution Of Symptoms	N/A
Chu And Chiang [44]	2003	49	Male	Ectopic Tooth	Nasal Cavity	Hard Painful Lesion In Right Nasal Floor	N/A	N/A	Endoscopic	N/A	Complete Resolution Of Symptoms	N/A

The clinical presentations of patients in these studies was varied, and Table 2 stratifies the location of the ectopic tooth by clinical presentation of the patient. Twelve patients (48.14%) had nasal obstruction, six patients (22.2%) had facial pain, six patients (22.2%) had rhinorrhea/nasal discharge, six patients (22.2%) had epistaxis, two patients (7.4%) had a foul odor sensation, one patient (3.7%) had nasal regurgitation, one patient (3.7%) had hyposmia, and one patient (3.7%) had hemoptysis.

**Table 2:** Symptoms described in relation to paranasal and nasal cavity teeth

Symptom	Number of Patients	Location of Tooth: Nasal Cavity	Location of Tooth: Paranasal sinus
Nasal Regurgitation	1	0	1
Hyposmia	1	0	1
Hemoptysis	1	0	1
Nasal Obstruction	12	10	2
Facial Pain	6	1	5
Rhinorrhea/nasal discharge	7	5	2
Epistaxis	6	6	0
Foul odor sensation	2	2	0

21 patients (78%) and 14 patients (52%), received a preoperative CT paranasal sinus scan and x-ray, respectively, for surgical planning and diagnostic purposes. Only three studies reported use of any postoperative imaging and all of these studies reported no pathology noted on imagining postoperatively. Data regarding the modality of imaging used and the patients’ preoperative endoscopic and imaging findings are included in Table 1.

All patients who were symptomatic had complete resolution of their symptoms following endoscopic surgery. No studies reported endoscopic findings following surgery. Only 12 studies reported the time that they followed their patients until after surgery. The time of follow-up averaged 8.5 months across these studies and ranged from 10 days to 2 years. (Table 1).

From a technical perspective, six of the studies described using a 0° endoscope for their approach. Two other studies used a 30° endoscope and another two studies used a 70° endoscope. The remaining studies did not specify what type of endoscope they used for visualization. Table 3 describes the specific operative technique used according to location of the nasal tooth. Fifty percent of maxillary sinus teeth were removed via a maxillary antrostomy. Eighty five percent of nasal cavity teeth were removed with simple extraction under endoscopic visualization.

**Table 3:** Operative Techniques Reported in Included Papers

Operative Technique Used For Tooth Removal	Number of Patients (Total)	Number of Patients per Location of Tooth: Nasal Cavity	Number of Patients per Location of Tooth: Paranasal Sinus
Maxillary Antrostomy with extraction	7	0	7
Uncinectomy	3	0	3
Curved Blakesley cup forceps extraction	2	1	1
Canine Fossa Bony Window with extraction	1	0	1
Elevator and negative suction pressure with extraction	1	0	1
Incision in nasal floor and avulsion with extraction	1	0	1
Simple extraction with endoscopic visualization under general anesthesia	11	11	0
Dislodged in incised mucosa.	1	1	0

## Discussion

Ectopic teeth in the nasal cavity and maxillary sinus, while reported in the literature, are not common [41, 1, 45]. Notably, there are very few cases in the otolaryngology literature. Supernumerary teeth only occur in 0.1–1% of the general population [17]. Despite a range of etiologies, the most common underlying reason for a maxillary sinus/nasal cavity foreign body is a history of a dental procedure [46]. We systematically reviewed the literature on the endoscopic removal of paranasal sinus and nasal cavity ectopic teeth. Previously, surgeons may have opted to remove such teeth via the Caldwell-Luc procedure. The more recent literature points to a shift towards endoscopic management of this pathology. At our own institution in the last year, two patients presented to our Otolaryngology clinic with ectopic paranasal sinus and nasal cavity teeth. Prior to removal, our first patient was experiencing symptoms of chronic rhinosinusitis including facial pain and rhinorrhea, as well as maxillary sinus opacification on a computed tomography (CT) scan, while the second patient complained mainly of nasal obstruction. We successfully completed endoscopic removal of a maxillary sinus tooth with chronic rhinosinusitis as well as the endoscopic removal of a nasal cavity tooth. Our systematic review showed a preponderance of males with this pathology. Interestingly, both of patients at our institution were male as well. A case series on ectopic teeth in the maxillary sinus also demonstrated the majority of patients were male [9, 47]. While there is no document pathophysiological reason for while males more likely present with ectopic sinonasal teeth, it may be important to consider from a clinical perspective.

Certain patterns emerged from this review regarding a correlation between clinical symptom and location of patient’s ectopic tooth. If the patient’s ectopic tooth was in their nasal cavity they were more likely to present with symptoms of nasal obstruction, rhinorrhea and epistaxis. The most common clinical symptom in paranasal sinus ectopic teeth was facial pain. One of the most common etiologies in ectopic paranasal/nasal cavity ectopic teeth is dental related trauma [46] and as such previous dental history should be elicited in patients with symptoms of nasal obstruction, rhinorrhea and epistaxis that are refractory to regular treatment. Additionally, a CT scan may be warranted for patients with these symptoms if previous dental history is present and they are refractory to their current treatment. The data in this review suggest that most surgeons ordered a pre-operative CT scan for their patients. CT findings included opacification of sinuses / thickening of mucosa in addition to the obvious tooth mass. Such CT findings are in keeping with patients’ clinical symptoms of chronic rhinosinusitis. X-ray films were also ordered by otolaryngologists, as well as referring dentists. These included orthopantrograms, Panorex views, Water’s views and occipitomental views. CT scan of the paranasal sinuses may be the most optimal modality for patient safety given its utility in identifying differing tissue types and allowing for surgical anatomical planning.

All patients who presented symptomatically had complete resolution of their symptoms following endoscopic removal of the ectopic tooth. Mechanistically, it is hypothesized that foreign bodies cause chronic rhinosinusitis due to constant mucosal irritation leading to infection and ciliary insufficiency [21, 48]. As such, once the instigating foreign body is removed, it is likely that the infection will resolve after appropriate irrigation and antibiotic treatment. Furthermore, it is generally recommended that foreign bodies in the maxillary sinus be removed even prior to symptom development [21]. It is also recommended that supernumerary nasal teeth be extracted early to prevent morbidity [3]. Both patients at our institutional also had clinical resolution of their symptoms after these surgical procedures. Despite reported resolution of symptoms in most patients, studies included in this review inconsistently reported their follow-up practices. Under half of the studies reported the time post-operatively that they re-evaluated their patients for signs of disease. Reported follow-up times had great variability, with some surgeons reporting follow-up of their patients 10 days after surgery and others until 24 months after surgery. Additionally, only three studies reported their patients’ resolution of disease with mention of any post-operative endoscopic or imagining evaluation completed. Post-operative endoscopic evaluation is important to ensure resolution, decide on need for additional antibiotics/topical treatment, as well as possible debridement.

We extracted data from the included studies regarding their preferred technique for ectopic tooth removal. Most of the studies used 0° nasal endoscopes while a minority used 30° and 70° nasal endoscopes. A 30-year review of endoscopic sinus surgery noted that the most commonly used endoscopes are 0, 30 and 70.^50^ There was no difference in outcome for patients depending on which angled endoscope was used as well as which approach, as all patients had complete resolution of their symptoms. The majority of the studies with patients with maxillary sinus teeth completed a middle meatal antrostomy / uncinectomy. The majority of studies with patients with nasal cavity teeth used some form of simple extraction or didn’t specify what their technique was. In general, in order to achieve retrieval of a tooth from a maxillary sinus endoscopically, the medial wall of the maxillary sinus must be opened widely. Grasping forceps such and angled pituitary or Heuwieser forceps or curettes are often used in the retrieval. Authors describing the nasal cavity teeth removals emphasized the importance of not damaging structures surrounding the tooth, such as nasal septal mucosa and cartilage. The required safety for extraction of nasal cavity teeth puts further importance on the visualization with the endoscopic.

Our systematic review presents with certain limitations. Primarily, there is limited research with a small sample size on the topic of endoscopic ectopic sinonasal teeth. As such, this encounters the issues commonly associated with limited powered studies, namely statistical confines and limited generalizability. Additionally, most of the research papers included in our review were case reports or case series. Some of these papers were missing data such as patients’ preoperative symptoms as well as specifics regarding the surgical technique. Thus, certain aspects of our analysis were limited.

Additional literature regarding endoscopic removal of ectopic sinonasal teeth vs the Caldwell-Luc approach would aid in management decision making, as well as adding to our understanding of the epidemiology and etiology of ectopic sinonasal teeth.

## Conclusion

While maxillary sinus and nasal ectopic teeth are uncommon, it is important for clinicians to consider this entity in the differential diagnosis. Our review demonstrates that there is scarce literature on endoscopic removal of maxillary sinus and nasal cavity teeth removal, specifically for patients presenting with symptoms of rhinosinusitis for whom endoscopic management may be preferred. With these case reports and review, we provide additional insight into the utility of endoscopic surgery as a safe and effective management strategy for ectopic maxillary sinus and nasal teeth.

## Data Availability

All data generated or analyzed during this study are included in this published article.

## Abbreviations

CT: Computed Tomography
MeSH: Medical subject headings
PRISMA: Preferred Reporting Items for Systematic Reviews and Meta-Analyses

## References

[1] Buyukkurt MC, Omezli MM, Miloglu O (2010). Dentigerous cyst associated with an ectopic tooth in the maxillary sinus: a report of 3 cases and review of the literature. Oral Surgery Oral Med Oral Pathol Oral Radiol Endod.

[2] Cai HX, Long X, Cheng Y, Li XD, Jin HX (2007). Dislocation of an upper third molar into the maxillary sinus after a severe trauma: a case report. Dent Traumatol.

[3] Kirmeier R, Truschnegg A, Payer M, Malyk J, Daghighi S, Jakse N (2009). The supernumerary nasal tooth. Int J Oral Maxillofac Surg.

[4] Amin ZA, Amran M, Khairudin A (2008). Removal of extensive maxillary dentigerous cyst via a Caldwell-Luc procedure. Arch Orofac Sci.

[5] Di Felice R, Lombardi T (1995). Ectopic third molar in the maxillary sinus. Case Rep Aust Dent J.

[6] Jude R, Horowitz J, Loree T (1995). A case report. Ectopic molars that cause osteomeatal complex obstruction. J Am Dent Assoc.

[7] Golden AL, Foote J, Lally E, Beideman R, Tatoian J (1981). Dentigerous cyst of the maxillary sinus causing elevation of the orbital floor. Oral Surg Oral Med Oral Pathol.

[8] Chuong R (1984). Dentigerous cyst involving maxillary sinus: report of case. J Am Dent Assoc.

[9] Vele DD, Sengupta SK, Dubey SP, Dokup MK (1996). Cystic lesions of the nasal cavity and the paranasal sinuses: report of two unusual cases. J Laryngol Otol.

[10] Altas E, Karasen RM, Yilmaz AB, Aktan B, Kocer I, Erman Z (1997). A case of a large Dentigerous cyst containing a canine tooth in the maxillary antrum leading to Epiphora. J Laryngol Otol.

[11] Nakamura N, Mitsuyasu T, Ohishi M (2004). Endoscopic removal of a dental implant displaced into the maxillary sinus: technical note. Int J Oral Maxillofac Surg.

[12] Chandrasena F, Singh A, Visavadia BG (2010). Removal of a root from the maxillary sinus using functional endoscopic sinus surgery. Br J Oral Maxillofac Surg.

[13] Sugiura N, Ochi K, Komatsuzaki Y (2004). Endoscopic extraction of a foreign body from the maxillary sinus. Otolaryngol Head Neck Surg.

[14] Oinam BS, Ningombam JS, Puyam SD, Thingbaijam S (2016). The role of endoscopic sinus surgery in maxillary sinus foreign body removal. J Med Soc.

[15] Brescia G, Saia G, Apolloni F, Marioni G (2017). A novel nasal endoscopic approach for removing displaced dental implants from the maxillary sinus. Am J Otolaryngol.

[16] Chagas Júnior OL, Moura LB, Sonego CL, de Farias EOC, Giongo CC, Fonseca AAR (2016). Unusual case of sinusitis related to ectopic teeth in the maxillary sinus roof/orbital floor: a report. Craniomaxillofac Trauma Reconstr.

[17] Dhingra S, Gulati A (2015). Teeth in rare locations with rare complications: an overview. Indian J Otolaryngol Head Neck Surg.

[18] Demirtas Nihat, Kazancioglu Hakki Oguz, Ezirganli Seref (2014). Ectopic Tooth in the Maxillary Sinus Diagnosed with an Ophthalmic Complication. Journal of Craniofacial Surgery.

[19] Biafora M, Bertazzoni G, Trimarchi M (2014). Maxillary sinusitis caused by dental implants extending into the maxillary sinus and the nasal cavities. J Prosthodont.

[20] Deniz Y, Zengin AZ, Karli R (2016). An unusual foreign body in the maxillary sinus: dental impression material. Niger J Clin Pract.

[21] Şahin YF, Muderris T, Bercin S, Sevil E, Kırıs M (2012). Chronic maxillary sinusitis associated with an unusual foreign body: a case report. Case Rep Otolaryngol.

[22] Beriat GK, Beriat NC, Yasinkaya E. Ectopic molar tooth in the maxillary sinus: a case report. Clin Dent Res. 2011;35(2):35–40.

[23] Academia and Clinic Annals of Internal Medicine (2009). Preferred Reporting Items for Systematic Reviews and Meta-Analyses.

[24] Al Nashawany M, Hassan OO, Ravi K, Alhumaid H, Taha EA, Parvez S, Sohbi M (2014). Endoscopic sinus surgery for the removal of foreign body (root) from the maxillary antrum: our experience. Case Rep Int.

[25] Hasbini AS, Hadi U, Ghafari J (2001). Endoscopic removal of an ectopic third molar obstructing the osteomeatal complex. Ear Nose Throat J.

[26] Di Pasquale P, Shermetaro C (2006). Endoscopic removal of a dentigerous cyst producing unilateral maxillary sinus opacification on computed tomography. Ear Nose Throat J.

[27] Saleem T, Khalid U, Hameed A, Ghaffar S. Supernumerary, ectopic tooth in the maxillary antrum presenting with recurrent haemoptysis. Head Face Med. 2010;6(1). 10.1186/1746-160X-6-26. 10.1186/1746-160X-6-26 PMC2992486 21070657

[28] Viterbo Stefano, Griffa Alessandro, Boffano Paolo (2013). Endoscopic Removal of an Ectopic Tooth in Maxillary Sinus. Journal of Craniofacial Surgery.

[29] Iwai Toshinori, Matsui Yoshiro, Hirota Makoto, Tohnai Iwai (2012). Endoscopic Removal of a Maxillary Third Molar Displaced Into the Maxillary Sinus via the Socket. Journal of Craniofacial Surgery.

[30] Clementini M, Morlupi A, Agrestini C, DI Girolamo M, DI Girolamo S, Ottria L (2012). Endoscopic removal of supernumerary tooth from the nasal cavity of a child: a case report. Oral Implantol (Rome).

[31] Nisa L, Giger R (2011). Images in clinical medicine. Ectopic tooth in the maxillary sinus. N Engl J Med.

[32] Chen A, Huang JK, Cheng SJ, Sheu CY (2002). Nasal teeth: report of three cases. Am J Neuroradiol.

[33] Aoki M, Jinbu Y, Matsumura T, Kusama M (2003). Ectopic tooth in the nasal cavity. Asian J Oral Maxillofac Surg.

[34] Kim DH, Kim JM, Chae SW, Hwang SJ, Lee SH, Lee HM (2003). Endoscopic removal of an intranasal ectopic tooth. Int J Pediatr Otorhinolaryngol.

[35] Lin I-H, Hwang C-F, Su C-Y, Kao Y-F, Peng J-P (2004). Intranasal tooth: report of three cases. Chang Gung Med J.

[36] Lee JH (2006). A nasal tooth associated with septal perforation: a rare occurrence. Eur Arch Oto-Rhino Laryngology.

[37] Verma RK, Bakshi J, Panda NK. Ectopic intranasal tooth: an unusual cause of epistaxis in a child. Ear Nose Throat J. 2012;91(6):242-4. 22711391

[38] Janardhan N, Kumar SR, Reddy RR, Kumar CA (2013). Rhinolithiasis due to supernumerary ectopic tooth: very rare case. Indian J Otolaryngol Head Neck Surg..

[39] Koosha M, Balasubramanian A, Mohamad I, Rahman WFWA (2014). Tooth in the nose. Bangladesh J Med Sci.

[40] Sanei-Moghaddam A, Hyde N, Williamson P (2009). Endoscopic removal of a supernumerary tooth from the nasal cavity in an adult. Br J Oral Maxillofac Surg.

[41] Larin RA, Kuzmin AV, Rylkin UA (2013). Endoscopic extraction of the impacted wisdom tooth from maxillary sinus. Sovrem Technol Med.

[42] Aydin U, Asik B, Ahmedov A, Durmaz A (2016). Osteoma and ectopic tooth of the left maxillary sinus: a unique coexistence. Balkan Med J.

[43] Ohki M (2012). Transnasal marsupialization using endoscopic sinus surgery for treatment of Keratocystic odontogenic tumor in maxillary sinus. Case Rep Otolaryngol.

[44] Chu CK, Chiang CW. Intranasal tooth: a case report. Tzu Chi Med J. 2003.

[45] Lim Daniel, Parumo Rosliza, Chai Ma Bee, Shanmuganathan Jothi (2017). Transnasal Endoscopy Removal of Dislodged Dental Implant: A Case Report. Journal of Oral Implantology.

[46] Agawal S, Kumar S (2014). Foreign bodies in maxillary sinus: causes and management. Astrocyte.

[47] Lai Y-TA, Luk YS, Fung K-H. Anomalous morphology of an ectopic tooth in the maxillary sinus on three-dimensional computed tomography images. J Radiol Case Rep. 2013;7(2). 10.3941/jrcr.v7i2.1227. 10.3941/jrcr.v7i2.1227 PMC3661307 23705035

[48] Pagella F, Emanuelli E, Castelnuovo P (1999). Endoscopic extraction of a metal foreign body from the maxillary sinus. Laryngoscope.

